# Renal replacement therapy is an independent risk factor for mortality in critically ill patients with acute kidney injury

**DOI:** 10.1186/cc9355

**Published:** 2010-12-01

**Authors:** Monique M Elseviers, Robert L Lins, Patricia Van der Niepen, Eric Hoste, Manu L Malbrain, Pierre Damas, Jacques Devriendt

**Affiliations:** 1 Department of Medicine, University of Antwerpen, Universiteitsplein 1, 2610 Wilrijk, Belgium; 2 Nephrology-Hypertension, University of Antwerpen, Universiteitsplein 1, 2610 Wilrijk, Belgium; 3 Nephrology-Hypertension, University Hospital Brussels, Laarbeeklaan 101, 1090 Brussels, Belgium; 4 Intensive Care Medicine, Ghent University Hospital, De Pintelaan 185, 9000 Gent, Belgium; 5 Intensive Care Medicine, ZNA Stuivenberg, Lange Beeldekensstraat 267, 2060 Antwerpen, Belgium; 6 Intensive Care Medicine, University Hospital Liège, Domaine Universitaire du Sart-Tilman, Bâtiment B35, 4000 Liège, Belgium; 7 Brugmann University Hospital, Place Arthur Van Gehuchten 4, 1020 Brussels, Belgium

## Abstract

**Introduction:**

Outcome studies in patients with acute kidney injury (AKI) have focused on differences between modalities of renal replacement therapy (RRT). The outcome of conservative treatment, however, has never been compared with RRT.

**Methods:**

Nine Belgian intensive care units (ICUs) included all adult patients consecutively admitted with serum creatinine >2 mg/dl. Included treatment options were conservative treatment and intermittent or continuous RRT. Disease severity was determined using the Stuivenberg Hospital Acute Renal Failure (SHARF) score. Outcome parameters studied were mortality, hospital length of stay and renal recovery at hospital discharge.

**Results:**

Out of 1,303 included patients, 650 required RRT (58% intermittent, 42% continuous RRT). Overall results showed a higher mortality (43% versus 58%) as well as a longer ICU and hospital stay in RRT patients compared to conservative treatment. Using the SHARF score for adjustment of disease severity, an increased risk of death for RRT compared to conservative treatment of RR = 1.75 (95% CI: 1.4 to 2.3) was found. Additional correction for other severity parameters (Acute Physiology And Chronic Health Evaluation II (APACHE II), Sequential Organ Failure Assessment (SOFA)), age, type of AKI and clinical conditions confirmed the higher mortality in the RRT group.

**Conclusions:**

The SHARF study showed that the higher mortality expected in AKI patients receiving RRT versus conservative treatment can not only be explained by a higher disease severity in the RRT group, even after multiple corrections. A more critical approach to the need for RRT in AKI patients seems to be warranted.

## Introduction

Acute kidney injury (AKI) occurs in up to 25% of critically ill patients admitted to the Intensive Care Unit (ICU) [1]. Despite well-established supportive care and technical advances in renal replacement therapy (RRT), mortality remains remarkably high in these patients. A review by YP Ympa and colleagues, including 80 studies covering 15,897 patients, revealed that mortality rates remained unchanged at around 50% over the last 50 years [2]. On the other hand, recent observations pointed to the relative decline of death rates attributable to AKI, despite a rise in the occurrence of AKI [3,4].

Conservative AKI treatment includes management of volume, electrolyte and acid-base homeostasis and specific drug management. Renal replacement therapy (RRT) is indicated for management of specific problems such as volume overload, hyperkalemia, acidosis and symptoms of uremia. However, hard data remain absent or conflictive regarding the timing to start dialysis [5]. Moreover, there is a consensus that RRT is life saving and not starting RRT will lead to death in severely ill AKI patients, but data are lacking to generalize this opinion. Research focused completely on the choice and the dose of RRT modality and particularly results of comparative studies between daily IRRT (intermittent hemodialysis) or CRRT (continuous veno-venous hemofiltration) remained a matter of debate during the last decades [6-8]. In recent years, several controlled studies [9-12] and meta-analysis [13,14] showed similar benefit with either dialysis modality. Critics of the published studies, however, pointed to shortcomings such as lack of power, selection bias and disregarding differences in disease severity [10,15-17].

Within the Stuivenberg Hospital Acute Renal Failure (SHARF) project, we developed and validated a specific severity scoring system for AKI [18,19]. In this new, large scaled, prospective study (SHARF 4), we used the SHARF score to correct for differences in disease severity comparing different treatment modalities in AKI patients admitted to the ICU. The SHARF 4 study included a randomized clinical trial part with results on the comparison between IRRT and CRRT reported elsewhere [20]. This paper will focus on the overall observational results, comparing ICU and hospital outcome of AKI patients with conservative therapy or either treated with intermittent or continuous dialysis techniques.

## Materials and methods

### Selection of centers

Belgian ICUs were invited to participate in the SHARF4 study if they belonged to a hospital with at least 600 beds having a chronic dialysis unit and if they performed RRT treatment in at least 30 AKI patients during the last year. They qualified for participation if both intermittent and continuous RRT techniques belonged to their common practice. A center questionnaire was sent to candidate centers in order to check qualifying criteria.

### Selection of patients

All adult patients consecutively admitted to the ICU and having a serum creatinine >2 mg/dl were included. Patients with pre-existing chronic renal disease, defined as a serum creatinine above 1.5 mg or with clearly reduced kidney size on ultrasound, were excluded. In all included patients, disease severity was defined by calculating the SHARF score [19] and patients were classified in one of the SHARF severity classes accordingly (SHARF <30, 30 to 60, >60).

### Allocation of treatment

The decision to treat conservatively or to start RRT was at the discretion of the responsible physician, taking into account the rules of good clinical practice in this field. Patients in need of renal replacement therapy were assigned to daily IRRT (intermittent hemodialysis during four to six hours daily) or CRRT (continuous veno-venous hemofiltration) after randomization or according to local practice, if one of the predefined contraindications for randomization was present. The techniques used to perform RRT were in agreement with the standard procedures of the participating hospitals [20].

### Data collection

Basic data collection included demographic data, cause and type of AKI, type of primary disease, body weight and length and daily serum creatinine levels. Parameters of the SHARF score were collected at the first day that the criteria of AKI were met. Overall severity was evaluated with the Acute Physiology And Chronic Health Evaluation II (APACHE II) score [21] and with the Sequential Organ Failure Assessment (SOFA) score [22] at admission of the ICU. Short-term outcome parameters studied were mortality, ICU and hospital length of stay (LOS) and renal function at hospital discharge. Renal function was estimated using the Cockroft and Gault formula (eGFR) and stages of chronic kidney disease were defined according to the NKF K/DOQI guidelines [23].

### Statistical analysis

The data analysis was performed using SPSS, version 12.0 (SPSS Inc, Chicago, Illinois, US).

Outcome parameters studied were hospital mortality, length of stay in ICU and hospital and renal function at hospital discharge. Descriptive, univariate analysis was performed on all parameters in order to find significant differences between different treatment groups using Student's *t*-test and Chi square test. Multivariate analysis was performed using logistic regression with mortality as the dependent outcome variable. Correction for severity of illness was performed using the SHARF score as a continuous variable, completed with the APACHE II and SOFA score. For subgroup analysis, selection was based on reported evidence that these subgroups included the most complicated patients showing the highest co-morbidity and mortality. Confounding factors were selected if they showed a significant difference in the comparison between treatment options and contribute effectively and independently to the observed outcome. Statistical significance was set at the 0.05 level (two-sided).

### Institutional review board

The protocol has been approved initially by the Ethics Committee of the Stuivenberg Hospital in Antwerp followed by the Ethics Committee of each participating center. A written informed consent has been obtained from each patient or his representative in case the patient was unconscious or intubated.

## Results

### Description of included centers and patients

Nine ICUs participated in the SHARF4 study. Four of them (Centers 1 to 4 in Table 1) recruited patients during the entire three-year study period (April 2001 to March 2004). One center only started in 2004 and in four centers participation ended early due to internal organizational problems (Other centers in Table 1).

**Table 1:** SHARF score, RRT and Mortality per center

Center	n	SHARF	RRT	Mortality
		mean (SD)	%	%
**1**	158	61.8 (24.3)	45.6	43.0
**2**	412	58.4 (31.3)	47.8	44.4
**3**	387	68.8 (27.3)	53.7	55.8
**4**	223	55.2 (28.3)	54.7	54.7
**Others**	123	69.2 (27.6)	41.5	53.7

RRT, renal replacement therapy; SHARF score, Stuivenberg Hospital Acute Renal Failure score

A total of 1,303 patients with AKI, consecutively admitted to the ICU, were included. Mean age was 64 (range 15 to 96), 63% were male. At baseline, the mean SHARF score was 62.3 (SD 28.9), APACHE II score 23.9 (SD 10.4) and SOFA score 9.2 (SD 3.9). Basic characteristics of the overall population with comparison between the groups with conservative and with RRT treatment are presented in Table 2.

**Table 2:** Patient characteristics and clinical parameters

	Total group	Conservative	RRT	*P-*value of difference
**Number**	*n *= 1,303	*n *= 653	*n *= 650	

**Age: mean (range)**	66 (15 to 96)	67 (16 to 93)	64 (15 to 96)	<.001

**Male**	63.1%	62.8%	63.5%	
**Female**	36.9%	37.2%	36.5%	0.754

**Type of AKI**				
Pre-renal	45.5%	58.4%	32.6%	
Renal	54.5%	41.6%	67.4%	<.001

**Specified cause of AKI**				
Acute tubular necrosis	89.6%	89.6%	89.6%	
Other	10.4%	10.4%	10.4%	0.997

**Setting of AKI**				
Medical	72.8%	72.2%	73.4%	
Surgical	27.2%	27.8%	26.6%	0.634

**Severity scores (mean (SD))**				
SHARF (baseline)	62.3 (28.9)	58.4 (28.4)	66.0 (29.0)	<.001
APACHE II (baseline)	23.9 (10.4)	22.5 (10.2)	25.2 (10.4)	<.001
SOFA (baseline)	9.2 (3.9)	8.5 (3.8)	9.9 (3.9)	<.001

AKI: acute kidney injury RRT: Renal Replacement Therapy SHARF score: Stuivenberg Hospital Acute Renal Failure score [19]

### Treatment modality offered

**Figure 1:**
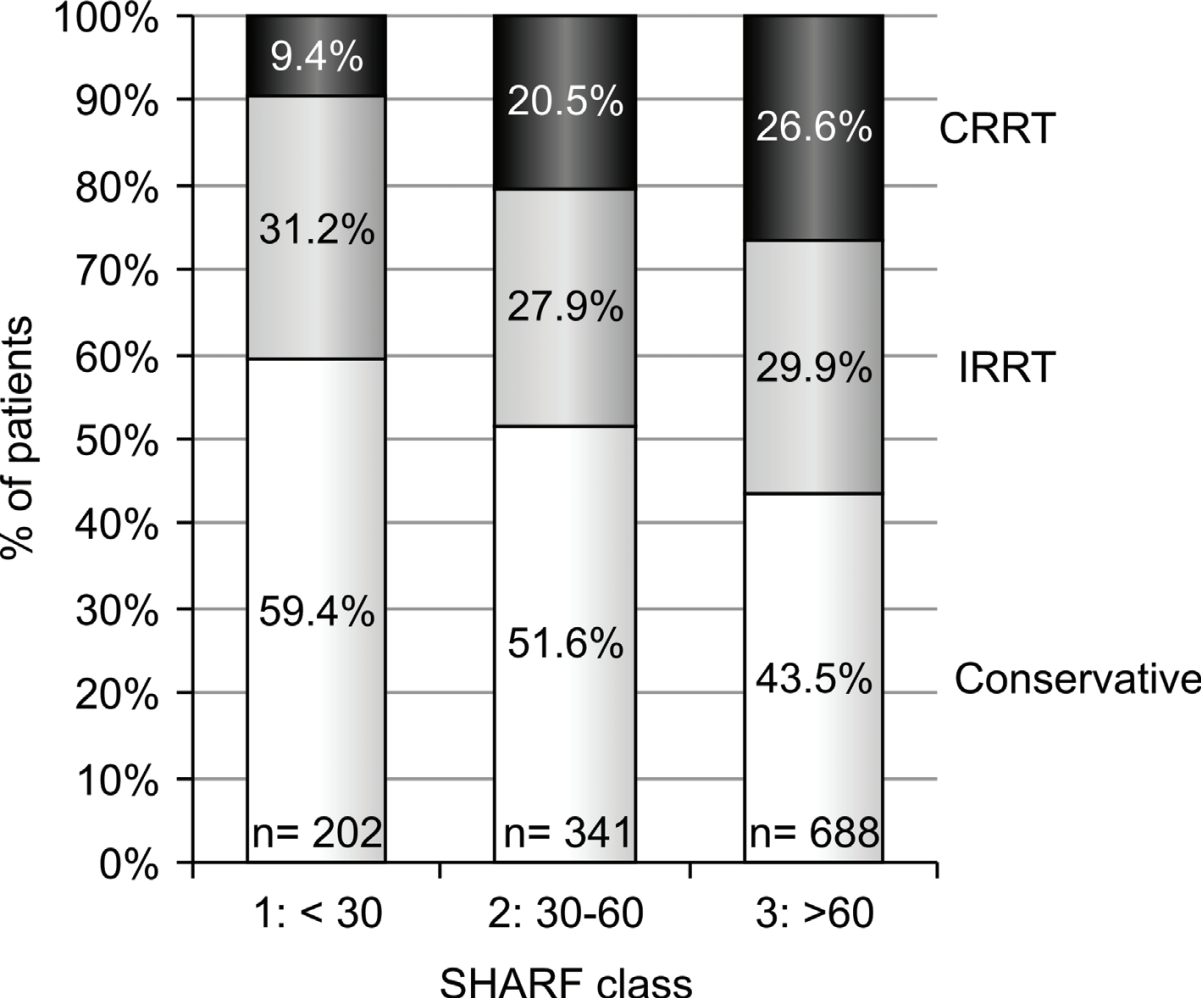
**Assignment to different treatment modalities within each SHARF score class**. CRRT, continuous renal replacement therapy; IRRT, intermittent renal replacement therapy.

RRT was initiated in 650 patients (49.9%). Among patients requiring RRT, 58% received IRRT and 42% received CRRT at their first day of treatment. Assignment to different treatment options differed significantly (*P *< .001) between the SHARF classes as shown in Figure 1. Within the highest SHARF class, relatively fewer patients were treated with conservative treatment and more with CRRT.

### Overall outcome in patients with AKI admitted to the ICU

During their hospitalization, 655 out of 1,303 patients died. Overall observed mortality was 50.3% ranging from 43 to 64% per center (Table 1). Within the three classes of the SHARF score, mortality increased from 22% in the lowest class to 64% in the highest class (Table 3).

**Table 3:** Outcome in total group and according to SHARF severity classes

	Overall	SHARF score			*P*-value of difference
		**<30**	**30-60**	**>60**	
**Number of AKI patients**	*n *= 1303	*n *= 202	*n *= 341	*n *= 688	
**Hospital mortality**	50.3%	21.8%	40.5%	63.7%	<0.001
**ICU and hospital stay**					
Days in ICU: mean (SD)	14.1 (16.4)	7.9 (10.0)	13.8 (16.2)	16.0 (17.4)	<0.001
Days in hospital: mean (SD)	34.2 (36.6)	29.0 (30.8)	38.8 (43.4)	33.4 (33.7)	0.009
**Renal outcome in survivors**					
CKD stage 1-2 (eGFR > = 60 ml/minute)	38.6%	30.7%	41.4%	43.2%	
CKD stage 3 (eGFR 30-59 ml/minute)	35.0%	34.3%	30.8%	39.2%	
CKD stage 4 (eGFR 15-29 ml/minute)	10.7%	12.4%	13.0%	7.0%	
CKD stage 5 (eGFR <15 ml/minute or ESKD)	15.7%	22.6%	14.8%	10.6%	0.009

AKI, acute kidney injury; CKD, chronic kidney disease; eGFR, estimated glomerular filtration rate; ESKD, end-stage kidney disease; SHARF score, Stuivenberg Hospital Acute Renal Failure score [19]

Mean ICU LOS was 14 days, mean hospital LOS was 34 days. Within the three classes of the SHARF score, mean ICU length of stay increased from 7.9 days to 16.0 days. At hospital discharge, patients had a mean eGFR of 66.6 ml/minute (SD 37.7) and eGFR was above 60 ml/minute (Chronic kidney disease (CKD) stage 1 to 2) in 39% of patients. On the other hand, 16% of patients were discharged while still being treated with RRT. They were considered as having developed end-stage kidney disease and started a chronic RRT program. CKD stage 5 at discharge was most frequently observed in the lowest SHARF class (Table 3).

### Comparative outcome in patients with conservative and RRT treatment

**Figure 2:**
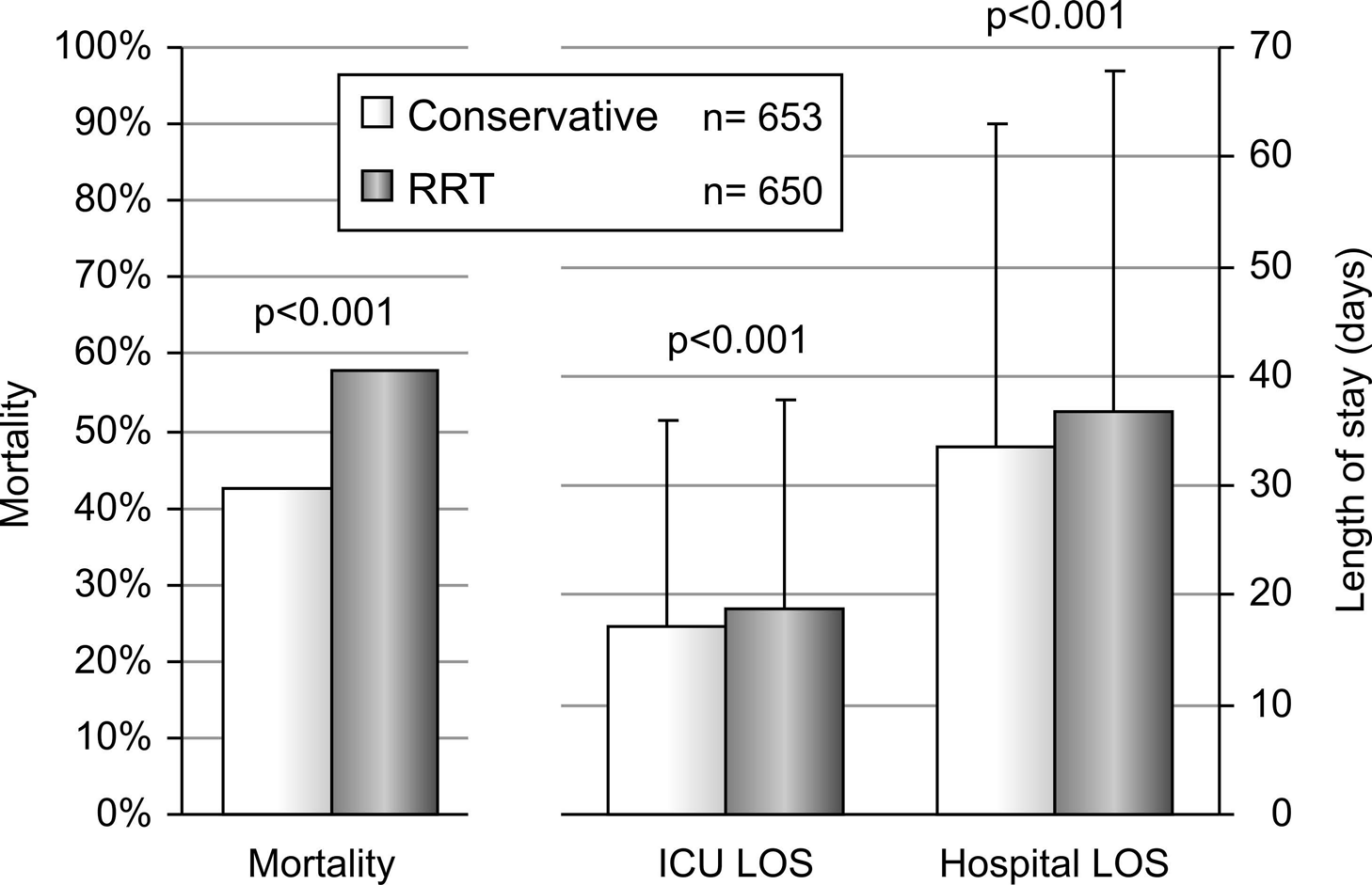
**Outcome in patients with conservative treatment and renal replacement therapy**. LOS, length of stay; RRT, renal replacement therapy.

**Figure 3:**
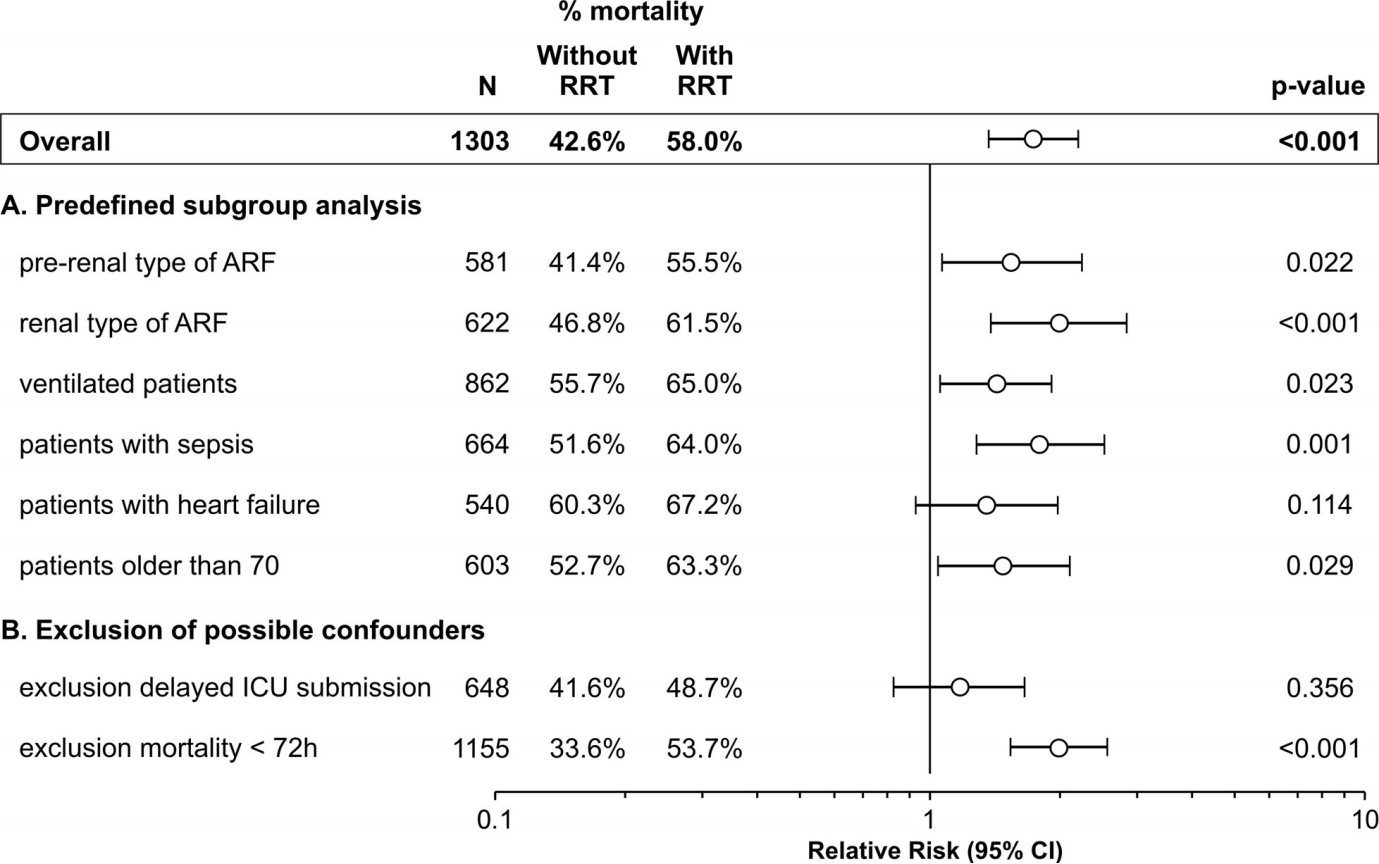
**Risk of mortality in patients with conservative treatment and renal replacement therapy**. Binary logistic regression analysis with 'without RRT' as reference category, controlled for disease severity using the SHARF score. **A**. Predefined subgroup analysis. **B**. Exclusion of possible confounders. AKI, acute kidney injury; ICU, intensive care unit; RRT, renal replacement therapy; RR (CI 95%), relative risk with 95% confidence interval.

AKI patients that were not treated with RRT showed an in-hospital mortality of 43% while patients with RRT had an in-hospital mortality of 58% (*P *< .001). Patients with RRT treatment showed a higher mortality as well as a longer ICU and hospital LOS (Figure 2). Even after a more in-depth correction for disease severity by using the individual SHARF scores in a logistic regression analysis, patients treated with RRT showed an increased risk of mortality of RR = 1.73 (95% CI: 1.4 to 2.2), This increased risk remained in subgroup analysis and after exclusion of possible confounders (Figure 3). Additional correction for confounding by introducing age and sex, other severity parameters (APACHE II, SOFA), type of AKI, delayed admission to the ICU and clinical conditions (ventilation, sepsis, heart failure) into the model, did not alter these results.

In survivors, at hospital discharge, an eGFR of less than 15 mL/minute (CKD stage 5) was observed in 9% of patients without RRT compared to 24% in patients treated with RRT (*P *< 0.001).

### Comparative outcome in patients treated in different centers

As shown in Table 1, large inter-center differences were observed in mean SHARF scores (*P *< .001) as well as in mortality (*P *= .003). Particularly in the SHARF 3 class (that is, patients with the highest disease severity) inter-center difference in mortality was most pronounced ranging from 48% to 76% (*P *< .001). RRT frequency however, did not correspond with mean disease severity per center. For example, center 1 had the lowest SHARF score and the highest dialysis frequency.

The influence of center practice with regard to the initiation of RRT on mortality is shown in Table 4. For this analysis, centers were ranked according to their RRT frequency with the lowest taken as reference center. Since the 'other' centers showed a wide variation in RRT treatment, this group was excluded from this analysis. While controlling for individual disease severity and treatment modality offered (conservative versus RRT), an increasing center risk of mortality was observed with increasing use of RRT (overall center influence *P *= .032) reaching a OR = 1.9 (95% CI: 1.2 to 2.9) in the center with most RRT treatment.

**Table 4:** Risk of mortality according to center of treatment

Center influence	n	% RRT	% Mortality	RR (95% CI)*
center 1	158	45.6	43.0	ref
center 2	412	47.8	44.4	1.2 (0.8 to 1.8)
center 3	387	53.7	55.8	1.4 (0.9 to 2.1)
center 4	223	54.7	54.7	1.9 (1.2 to 2.9)

* Overall *P *= .03

Binary logistic regression analysis with centers ranked according to their frequency of RRT treatment offered, with the center showing the lowest frequency of RRT taken as reference category. Center risk was controlled for individual treatment offered (conservative versus RRT) and individual disease severity (SHARF score).

RR (95% CI), relative risk with 95% confidence interval; RRT, renal replacement therapy.

## Discussion

In this multi-center SHARF4 study, including 1,303 consecutively admitted AKI patients, we found significant differences in outcome between patients receiving conservative treatment and those treated with RRT. Prognosis of RRT patients remained worse, after correction for disease severity or limiting the analysis to the most critically ill patients. Center practice of treatment choice was identified as an independent risk factor for mortality, with the higher frequency of RRT treatment associated with higher mortality.

Although our results may be due to differences in severity of disease in general and renal failure in particular, no guidelines were available to define this severity more accurately. The more recently introduced RIFLE criteria [24] were not yet validated during the study period [25-27]. We also have no arguments to suspect that our results are related to the quality of dialysis treatment on itself. Taking into account, the large inter-center variation in the decision to start RRT treatment irrespective of the SHARF score, it will be very difficult to obtain more conclusive results, particularly based on observational study designs.

There is still insufficient data to determine absolute indications and optimal timing for initiation of RRT in patients with AKI. In some patients, early start of renal support may improve outcome. However, early initiation may expose other patients unnecessarily to the risks of RRT [5]. The AKI Network reviewing the evidence in this field, stated that 'the indications for RRT must be viewed within the context of the patient's entire clinical condition with most indications being relative and only a small number of absolute indications' [28].

Although well-established recommendations about initiation of RRT in AKI patients are lacking, one should at least expect to find some outcome research in this field. It seems, however, that conservative treatment for AKI has so far only been considered as the treatment option for less severe patients. It was never considered as a meaningful alternative treatment, worthwhile to be included in research projects comparing outcome in different treatment modalities for AKI. In this regard, the recently published observations of the VA/NIH Trial are of particular interest [29]. This clinical trial revealed that intensive renal support in critically ill AKI patients did not decrease mortality or improve renal recovery compared with less intensive therapy.

In our study, the initiation of RRT was at the discretion of the responsible physician, taking into account the rules of good clinical practice in this field. It looks however that the balance between the advantages and disadvantages of starting RRT treatment was interpreted in a different way in the ICUs participating in the SHARF study. The difference in center practice is clearly demonstrated in Table 1 showing no relationship between the mean SHARF score per center and the percentage of patients treated with RRT. This observation corroborated the more generalized statement of the AKI Network that the provision of RRT in AKI patients is extremely variable and based primarily on empiricism and local institutional practice and resources [28]. The AKI Network, as well as the Acute Dialysis Quality Initiative (ADQI) Group two years earlier, emphasized the high need of additional evidence in this field based on well-designed trials and observational studies [24,28].

Additionally, cost considerations can also play a more pronounced role in the decision-making process in the future. For patients with uncomplicated AKI, it has been demonstrated that dialysis therapy was one of the most prominent factors independently associated with direct hospital costs and hospital LOS [30].

The clinical trial part of this SHARF 4 study corroborated the conclusion that benefit with either dialysis modality could not be observed [20]. Additional comparison within and between both treatment options in respect to delivered dose showed no effect on outcome [31], as recently also confirmed by a meta-analysis [32]. Since evidence is growing about comparable outcome in both modalities [10-12,33], also consensus is growing to merely consider both treatment options as complementary. On the one hand, there is the opinion that both techniques can be used interchangeably in critical ill AKI patients, according to circumstances [34]. Others stressed that both methods are complementary with IRRT for faster elimination of electrolytes and waste products and CRRT for regulation of higher calories requirements and for hemodynamically unstable patients [16]. Additionally, it has been mentioned that, although both treatments have a similar outcome, one or both has an absolute preference in specific conditions such as IRRT in patients with specific bleeding risk or CRRT in patients with cerebral edema or liver failure [5].

Recently, two retrospective cohort studies confirmed the equal outcome for mortality but revealed a better renal recovery in patients treated with CRRT [35,36]. We observed the same trend with 28% of IRRT patients compared to only 18% of CRRT patients with an eGFR of less than 15 mL/minute (stage 5) at hospital discharge (*P *= .107). Questions remain, however, if the eGFR at hospital discharge can be considered as the outcome of renal function after AKI. Although at the time of this study no consensus existed about the optimal timing to evaluate definitively the renal outcome after AKI, the presented classification can only be considered as a preliminary result. A mean hospital LOS of 34 days together with the skewed distribution of this parameter (range 1 to 339 days) hampered a definitive classification. Indeed, in our long-term follow-up study of hospital survivors of this cohort, we observed that 13 out of the 27 patients considered as ESKD at hospital discharge became dialysis independent, while 7 patients went on to need chronic dialysis treatment within the first year after hospital discharge [37].

In this study, we tried to formulate our conclusions carefully, only stressing the need to re-consider the value of conservative treatment as a valid and independent option in the treatment of AKI. We are aware about the limitations of our results based on an observational study design. Particularly concerns arise about the 'between' and 'within' homogeneity of patients with conservative treatment and RRT, as well as about their equal eligibility for RRT initiation in view of disease severity. Despite our attempts to control for bias, including all available and possible confounders in the multi variable model, a number of well designed clinical trials will be needed to obtain more definitive conclusions.

## Conclusions

This cohort study of 1,303 AKI patients consecutively admitted to the ICU confirmed that mortality is equal in patients treated with intermittent or continuous RRT. However, prognosis was significantly worse in those receiving RRT compared to conservative treatment and this difference remained significant after correction for the severity of disease and in different subgroup analysis. A higher mortality was observed in centers with a higher frequency of RRT treatment. As the indication for RRT differs between centers and between individual physicians, this conclusion needs to be validated in further prospective studies using evidence-based standards for the indication and timing to initiate RRT. Meanwhile, and in line with other recent observations, an integrated and individualized approach, considering conservative management as well as different RRT options in each patient, seems to be warranted.

## Key messages

• In this cohort study of 1,303 AKI patients consecutively admitted to the ICU, prognosis was significantly worse in those receiving RRT compared to conservative treatment.

• The higher mortality in AKI patients receiving RRT versus conservative treatment remained significant after multiple corrections for severity of disease and in different subgroups, thus can not only be explained by a higher disease severity in the RRT group.

• Within the group of RRT patients, this study confirmed that mortality was equal in patients treated with intermittent or continuous RRT.

• An individualized approach, integrating conservative management as well as different RRT options in each patient, deserves more attention.

• Center policy regarding the starting of RRT in AKI patients admitted to the ICU differed widely in Belgium.

## Abbreviations

AKI: acute kidney injury; APACHE II score: Acute Physiology and Chronic Health Evaluation II score; CRRT: Continuous Renal Replacement Therapy; eGFR: estimated glomerular filtration rate; IRRT: Intermittent Renal Replacement Therapy; LOS: Length Of Stay; RRT: Renal Replacement Therapy; SHARF score: Stuivenberg Hospital Acute Renal Failure score; SOFA score: Sequential Organ Failure Assessment score.

## Competing interests

The authors declare that they have no competing interests.

## Authors' contributions

MME conceived of the study design, analysed and interpretated data, and drafted and revised the article. RLL, PVdN, MLM, EH, PD and JD conceived of the design, analysed and interpretated data, and drafted and revised the article. All authors provided intellectual content of critical importance to this project and gave their final approval of this version to be published.
